# Psychological and physiological moderators of perceived exertion in aerobics: a repeated-measures study

**DOI:** 10.3389/fpsyg.2026.1719976

**Published:** 2026-05-07

**Authors:** Xiaoxue Gao, Jincheng Zhao, Tongkai Zhao, Yong-Gwan Song, Yuyao Zhi, Xinyi He

**Affiliations:** 1 Pukyong National University, Busan, Republic of Korea; 2 Xinjiang University of Science and Technology, Korla, China; 3 Party School of the CPC Yanjin County Committee, Xinxiang, Henan, China; 4 Chengdu Sport University, Chengdu, China

**Keywords:** aerobics, perceived effort, physical exercise, physical fitness, psychological traits

## Abstract

**Objectives:**

Perceived exertion is a central marker of exercise intensity, yet individuals differ substantially in how hard a standardized workload feels. This study examined whether anthropometric, physiological, and psychological characteristics moderate the association between achieved heart rate reserve (HRR) and perceived exertion during standardized step-aerobics.

**Methods:**

A total of 126 young adults (18–35 years; 63 females, 63 males) completed three step-aerobics sessions targeted to light, moderate, and vigorous intensities (40%, 60%, and 75%–80% HRR). Heart rate and rating of perceived exertion (RPE; Borg 6–20) were recorded every 3 min, yielding 1,134 repeated observations. Baseline assessments included anthropometry, bioimpedance-derived body composition, Cooper-test–estimated cardiorespiratory fitness, and psychological questionnaires assessing exercise self-efficacy, BIS/BAS, trait anxiety, interoceptive sensibility (MAIA-2), intensity preference, and intensity tolerance. Repeated-measures generalized estimating equation models tested prespecified HRR × moderator interactions, and exploratory models examined session-specific MAIA-2 effects and the HRR × MAIA-2 × sex interaction.

**Results:**

Perceived exertion increased strongly with HRR overall (B = 0.167, 95% CI [0.157, 0.176], standardized *β* = 0.859, *p* < 0.001), indicating a large and practically meaningful coupling between internal load and perceived exertion. None of the prespecified physiological moderators significantly altered the HRR–perceived-exertion slope, and the interaction effects were small, suggesting limited practical relevance of physiological moderation once exercise was standardized by relative HRR. Psychological moderation effects were also small; the largest estimate was observed for MAIA-2 (HRR × MAIA-2: B = −0.0078, 95% CI [−0.0160, 0.0003], standardized *β* = −0.040, *p* = 0.060). In exploratory session-specific models, a 1-SD higher MAIA-2 score was associated with a 0.18-point lower Borg RPE at moderate intensity (B = −0.176, 95% CI [−0.348, −0.003], standardized *β* = −0.106, *p* = 0.046), indicating a statistically detectable but small absolute difference in perceived exertion. The HRR × MAIA-2 × sex interaction was not significant (B = 0.011, 95% CI [−0.004, 0.026], standardized *β* = 0.058, *p* = 0.137). During vigorous exercise, RPE values clustered near the upper end of the Borg scale, consistent with a perceptual ceiling effect.

**Conclusion:**

When step-aerobics is standardized by relative internal load, achieved HRR is the dominant determinant of perceived exertion. Physiological status contributes little additional moderation, whereas interoceptive sensibility showed only limited exploratory evidence of a small association with lower perceived exertion at moderate intensity.

## Introduction

Perceived exertion is the conscious sense of how hard, heavy, and strenuous exercise feels, and it is widely used as a practical indicator of exercise intensity (Scherr et al., 2013). Across exercise modalities, ratings of perceived exertion generally increase in parallel with physiological load, supporting their value for both research and applied prescription (Lea et al., 2022). At the same time, individuals often differ substantially in how hard the same relative workload feels, suggesting that the perceptual response to exercise may be shaped by factors beyond momentary cardiovascular strain (Grummt et al., 2024).

Step-aerobics provides a useful context in which to study this question because the external task can be standardized while internal responses still vary across participants (Darby et al., 1995). In bench/step exercise, energy cost and cardiorespiratory demand are known to change with step height, cadence, and movement characteristics, making this modality both ecologically relevant and experimentally controllable (Scharff-Olson et al., 1996). This combination makes step-aerobics well suited for examining why participants exposed to a similar choreographed stimulus may nevertheless report different levels of perceived exertion (Stanforth et al., 1993).

Despite these controllable external loads, people often report very different levels of “how hard it feels” at the same nominal intensity. Physiological status is a prime source of this variability. Evidence suggests that considering body composition alongside fitness can sharpen the interpretability of perceived exertion and endurance capacity, with skeletal muscle mass and percent fat offering meaningful context for the sensation-performance link (Lichti et al., 2023). In parallel, a meta-analysis comparing normal-weight versus overweight/obese groups indicates systematic differences in perceived exertion at matched intensities, moderated by age, exercise type, and environmental conditions—implicating adiposity as a plausible moderator of the exertional experience (Yu et al., 2021). Beyond momentary sensation, perception of exercise difficulty carries behavioral relevance: among formerly overweight women, higher perceived difficulty during a submaximal test predicted one-year weight regain, hinting that trait-like or stable factors bias exertional appraisal with downstream consequences (Brock et al., 2010).

Psychological dispositions also shape how intense a given workload feels. In adolescents, behavioral inhibition (BIS) was associated with lower tolerance for high intensity and more negative affect during both moderate and hard exercise, whereas behavioral activation (BAS) aligned with greater enjoyment and more positive affect—patterning how effort is evaluated as intensity rises (Schneider and Graham, 2009). Stable individual differences in intensity preference and tolerance are measurable with validated instruments such as the PRETIE-Q, which demonstrates sound internal structure and has been linked to chosen training levels in free-living exercise (Ekkekakis et al., 2008). Self-efficacy—confidence in one’s capacity to exercise—modulates the exertional trajectory itself: in sedentary adults, self-efficacy predicted both linear and quadratic changes in perceived exertion as workloads increased (Hu et al., 2007). Related work in youth shows lower pre-activity self-efficacy accompanying higher exertional ratings at a fixed relative intensity, suggesting reciprocal influences between confidence and how hard an equivalent task feels (Robbins et al., 2004). Interoceptive factors, the way people sense internal bodily signals, may further tilt effort appraisal. Good versus poor heartbeat perceivers differ in psychophysiological responses during strenuous exercise, implying that interoceptive accuracy can influence self-regulation of effort and perceived exertion, even if effects are modest and context-dependent (DGDS et al., 2019). At the same time, measurement matters: critiques of the heartbeat counting task caution that estimation strategies can contaminate “accuracy” scores, advocating for cautious interpretation and the use of multidimensional, validated self-reports such as the MAIA-2 when studying interoceptive traits in exercise settings (Desmedt et al., 2018; Mehling et al., 2018).

One plausible source of this variability is physiological status. Differences in fitness and body composition may influence how exercise is experienced, even when the task appears externally similar. Prior work suggests that adiposity, muscularity, and cardiorespiratory fitness can alter the relationship between physiological demand, endurance capacity, and perceived difficulty (Olson et al., 1991). These observations support the possibility that body-composition and fitness variables may moderate perceived exertion during standardized aerobics (da Cunha et al., 2011; Schantz et al., 2019). Precisely because step-aerobics combines ecological validity with strong experimental control, it provides a particularly suitable model for testing whether between-person differences in perceived exertion reflect not only physiological status, but also psychological dispositions under the same choreographed stimulus.

To our knowledge, prior studies have not simultaneously examined whether cardiorespiratory fitness, adiposity/body composition, and trait-level psychological characteristics explain between-person variation in RPE during the same standardized step-aerobics protocol adjusted by relative heart-rate reserve. Existing work has typically emphasized physiological demand in bench/step exercise or isolated psychological constructs in other exercise modes, rather than integrating these moderator domains within one repeated-measures framework (Scharff-Olson et al., 1996). Moreover, although heart-rate–based control can individualize aerobic workloads, the precision with which heart-rate reserve methods approximate ventilatory domains can vary with fitness and context, reinforcing the need to treat observed heart-rate responses as covariates when modeling perception. Historically, this approach stems from the heart-rate reserve concept introduced in Karvonen’s seminal longitudinal work, which still informs field prescriptions and offers a practical bridge between external choreography and internal load in aerobics research (Karvonen et al., 1957).

Against this backdrop, the present study is designed to clarify how both physiological status and psychological traits moderate perceived exertion during standardized step-aerobics sessions calibrated by heart-rate reserve. The novelty of the study lies in jointly modeling anthropometric/body-composition, cardiorespiratory-fitness, and psychological moderators within the same repeated-measures, HRR-calibrated step-aerobics design. This rationale is consistent with contemporary psychophysiological and psychobiological accounts of perceived exertion, which conceptualize effort perception as a conscious percept emerging from the integration of exercise-related bodily strain with central cognitive-affective appraisal; under this framework, both physiological status and trait-level psychological factors may plausibly moderate RPE at a given relative load (Lopes et al., 2022; Pageaux, 2014). Our primary hypothesis was that physiological status would moderate the HRR–RPE association during step-aerobics. Specifically, we hypothesized that higher field-estimated cardiorespiratory fitness (VO₂max from the Cooper 12-min run test) would be associated with a shallower increase in RPE across exercise intensities, whereas greater adiposity (body mass index, waist-to-height ratio, and fat mass from bioelectrical impedance analysis) would be associated with a steeper increase in RPE after accounting for relative HRR. Our secondary hypothesis was that psychological traits would also moderate the RPE response. We hypothesized that greater exercise self-efficacy (EXSE), stronger behavioral activation (BAS), higher preference/tolerance for intensity (PRETIE-Q), and greater interoceptive sensibility (MAIA-2) would be associated with lower RPE values at a given HRR, whereas higher behavioral inhibition (BIS) and greater trait anxiety (STAI-Y2) would be associated with higher RPE values at the same physiological load. In addition, we conducted exploratory analyses to examine whether any MAIA-2 association varied by prescribed intensity and whether the HRR–RPE association differed according to the combined effect of MAIA-2 and sex. Because these analyses were exploratory, no directional *a priori* hypotheses were specified for them.

## Methods

### Study design

**Figure 1:**
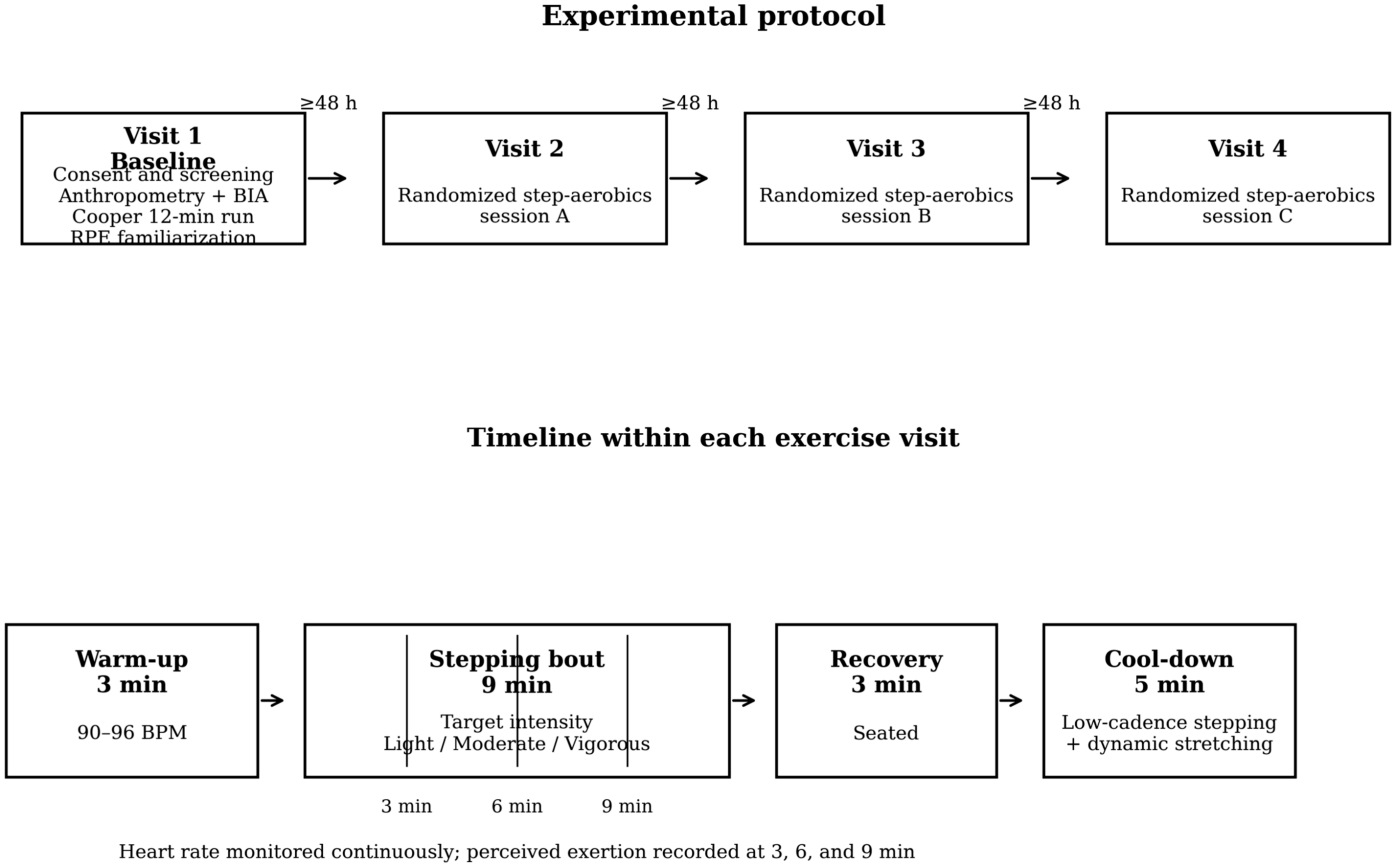
Schematic overview of the experimental protocol.

This research was conceived as a repeated-measures experimental study with a within-subjects design (Figure 1). Each participant completed three separate step-aerobics sessions corresponding to light, moderate, and vigorous intensities, with the order of sessions randomized and counterbalanced across participants so that the possible condition sequences were distributed as evenly as feasible, thereby minimizing sequencing effects. Intensities were individualized using the heart-rate reserve (HRR) method, derived from resting heart rate and maximal heart rate obtained during a field fitness test. The experimental structure allowed for the evaluation of within-person changes in ratings of perceived exertion (RPE) across standardized aerobic workloads while simultaneously modeling between-person moderators, including cardiorespiratory fitness, body composition, and psychological traits.

All experimental sessions were conducted in an indoor fitness studio equipped with step platforms of fixed height (20 cm) and a calibrated metronome or music tracks to control cadence. To minimize pacing variability, all tempos were preselected in advance, delivered using the same audio system, and kept constant within each 9-min exercise bout; tempo was not adjusted manually once a bout had started. The environment was maintained between 20 °C and 22 °C with adequate ventilation and space to accommodate stepping activity safely. Two certified exercise professional supervised all sessions, ensuring correct execution of step patterns, adherence to cadence, and continuous monitoring of heart rate.

The study involved four visits. At baseline, participants provided informed consent, completed eligibility screening, and underwent initial assessments, including body composition using multi-frequency bioelectrical impedance analysis (BIA) and the Cooper 12-min run test performed on a marked athletics track. The run test required participants to cover as much distance as possible within 12 min, and maximal oxygen uptake (VO₂max) was estimated using the validated regression equation associated with the distance achieved. Participants were also familiarized with the Borg 6–20 scale and practiced reporting RPE during a short stepping trial. To evaluate potential learning effects, RPE responses from the familiarization trial were compared with the first experimental session, and coefficient of variation was 1.9%. The following three visits consisted of the experimental exercise sessions. Each session included a standardized warm-up, one 9-min bout of step aerobics at the prescribed intensity (light, moderate, or vigorous), a 3-min seated recovery, and a cool-down period. Heart rate was recorded continuously throughout, and RPE was assessed every 3 min and at the end of each bout. Sessions were scheduled at least 48 h apart to minimize residual fatigue and ensure recovery.

### Participants

An *a priori* sample size estimation was conducted in G*Power 3.1, modeling our primary test as a repeated-measures ANOVA within–between interaction (three within-person intensity levels × a continuous between-person moderator) (Faul et al., 2007, 2009; Kang, 2021). We used a conservative small interaction effect of *f* = 0.12 (η^2^ = 0.014), *α* = 0.05 (two-tailed), and 1 − *β* = 0.80. This choice was made because the study was powered for moderator interaction effects, which are typically harder to detect than main effects and often modest in magnitude. The value is also close to the conventional small-effect ANOVA benchmark (Brookes et al., 2004; Lakens, 2013). The correlation among repeated measures was set to r = 0.60 and the nonsphericity correction to *ε* = 0.75, consistent with published test–retest reliability of RPE and session-RPE across visits in exercise settings (Crawford et al., 2018; Lamb et al., 1999). Under these assumptions, G*Power returned a minimum total sample size of ~100 participants for the within–between interaction; allowing for 15%–20% attrition due to missed sessions or unusable heart-rate data, we set a target enrollment of N = 120 to retain ≥80% power for the primary interaction tests in the final analytic sample.

Participants were recruited through convenience sampling using flyers, social media announcements, and word-of-mouth within university campuses, fitness centers, and community sports clubs in the city. Recruitment materials described the study as an investigation of “exercise perception during aerobics,” without emphasizing specific hypotheses, in order to minimize expectancy effects. Interested individuals contacted the research team and underwent an initial screening via online form. Eligible volunteers were then invited to the laboratory for baseline assessments, where written informed consent was obtained prior to data collection. The sampling strategy aimed to capture a heterogeneous but healthy adult cohort between 18 and 35 years, encompassing both sexes and a range of physical activity backgrounds, consistent with prior work examining moderators of perceived exertion in field and laboratory exercise settings (Scherr et al., 2013).

Eligibility for participation was restricted to adults aged 18–35 years in order to limit age-related variance in physiological capacity. Participants must be apparently healthy as determined via the PAR-Q + tool—a validated screening instrument widely used to identify risks associated with physical activity participation (Bredin et al., 2013). Individuals must not have a diagnosed cardiovascular, pulmonary, metabolic, or chronic disease, nor be under medication that substantially alters heart-rate response (e.g., *β*-blockers). Pregnancy and current musculoskeletal injury that would impair running or stepping movement were exclusionary, as was any acute illness in the prior 2 weeks. To assure the validity of fitness estimates, exclusion also applies to persons unable to complete the Cooper 12-min run test under standard conditions. The Cooper test has been shown to be valid and reliable for estimating VO₂max in healthy young adult populations, as well as to have strong test–retest reliability and criterion validity versus laboratory measurement (Bandyopadhyay, 2014; Penry et al., 2011).

A total of 126 participants (63 males, 63 females) aged 18 to 35 years completed the baseline evaluations. The overall mean age was 26.2 years (SD = 5.0), with no meaningful difference between sexes. Mean stature was 175.8 cm (SD = 7.5) in males and 165.2 cm (SD = 6.3) in females, and average body masses of 77.2 kg (SD = 10.8) and 63.4 kg (SD = 8.9), respectively. Mean body mass index averaged 25.0 kg·m^−2^ (SD = 2.9) in males and 23.2 kg·m^−2^ (SD = 2.7) in females. Waist circumference averaged 82.7 cm (SD = 7.8) in males and 74.5 cm (SD = 7.1) in females, yielding a waist-to-height ratio of 0.47 (SD = 0.06) and 0.45 (SD = 0.07), respectively. Males presented lower body fat percentage (18.4 ± 4.2%) and higher skeletal muscle mass (35.9 ± 5.7 kg), while females showed higher fat percentage (24.6 ± 4.6%) and lower skeletal muscle mass (28.2 ± 4.1 kg). Fat mass averaged 14.3 kg (SD = 4.1) in males and 15.7 kg (SD = 3.8) in females.

All study procedures were designed and conducted in accordance with the ethical principles of the Declaration of Helsinki, which provides internationally recognized guidelines for research involving human participants. Prior to participation, all volunteers received detailed written and verbal information about the study aims, procedures, risks, and benefits, and provided written informed consent. Ethical approval was obtained from the institutional research ethics committee of Chengdu Sport University with the code number (2025)114, which reviewed and authorized the protocol before recruitment commenced. Participants were assured of confidentiality and the right to withdraw from the study at any time without penalty.

### Intervention sessions

Each participant completed three intervention sessions consisting of structured step-aerobics exercise performed at light, moderate, and vigorous intensities. Sessions were carried out in an indoor studio maintained at 20–22 °C, with standardized flooring and ventilation, and were supervised by a certified exercise professional. To minimize carry-over fatigue, each session was separated by at least 48 h, and the order of intensities was randomized within a counterbalanced allocation scheme across participants.

All sessions followed a fixed timeline of approximately 20 min. Participants began with a 3-min warm-up performed to a music tempo of 90–96 beats per minute (BPM), corresponding to a cadence of 22–24 step cycles per minute (one step cycle = up with right foot + up with left foot + down with right foot + down with left foot). The warm-up consisted of marching on the floor, alternating side steps, and basic step-ups without arm movements. This was followed by a 9-min exercise bout at the assigned target intensity, structured as three consecutive 3-min blocks. After the exercise bout, participants completed a 3-min seated recovery in a chair, and then a 5-min cool-down, consisting of low-cadence stepping (~96 BPM), side taps, and light dynamic stretching of the quadriceps, hamstrings, and calves.

Step height was held constant at 20 cm for all participants to stabilize external load, while music tempo (in beats per minute, BPM) was manipulated to reach individualized relative intensity levels using heart-rate reserve (HRR). Before the experimental sessions, each participant completed a cadence-calibration ramp in which stepping was performed at prespecified BPM levels using the same standardized step height and movement pattern. Heart rate was recorded continuously, and the mean HR during the final portion of each stage was used to estimate the corresponding %HRR at that cadence. The target cadence for each intensity condition was then selected as the BPM producing the value closest to the prespecified HRR zone (light: ~40%; moderate: ~60%; vigorous: ~75%–80%), with preference given to cadences that kept participants within ±5 beats·min^−1^ of the target zone during the subsequent session. If two adjacent cadences produced similar HR responses, the lower cadence was selected for safety and movement standardization. To reduce tempo-related variability, each target cadence was delivered using preselected music files with fixed BPM, played on the same sound system and at the same volume range across sessions. The same tempo file was maintained throughout each 3-min block, and no *ad hoc* instructor-driven tempo changes were made during the exercise bout.

Drawing on data from bench-step and cadence studies, more realistic BPM ranges are Light = 100–110 BPM, Moderate = 120–130 BPM, and Vigorous = 130–145 BPM, recognizing that the upper end may be tolerable only for fitter individuals. For example, in a bench-stepping study of aerobic dance routines, cadences of 125–130 BPM with moderate bench height elicited considerable cardiovascular and RPE responses in adults (Grier et al., 2001). Also, cadence thresholds of ~100 steps/min and ~130 steps/min have been validated as heuristic markers for moderate vs. vigorous absolute intensity in ambulatory and stepping contexts (McAvoy et al., 2021; Tudor-Locke et al., 2019).

To reduce inter-participant heterogeneity during cadence assignment, participants were provisionally grouped into fitness tertiles based on the Cooper 12-min run test solely as a practical calibration aid, so that those within each fitness level performed feasible absolute cadences while still attaining their prescribed relative HRR targets. These tertiles were used only to guide safe and realistic tempo selection and were not entered as factors in the main statistical models. This ensured that cadence assignments were feasible across the fitness spectrum and that vigorous sessions did not rely on unmanageably high tempos for lower-fitness individuals.

The movement repertoire was standardized but varied slightly across intensities to match cadence and maintain ecological validity. At light intensity, participants performed only basic step-ups (right/left lead), side steps, and marching on the platform, without arm involvement. At moderate intensity, the choreography included basic step-ups with alternating knee lifts, side leg lifts, and repeater steps (3 consecutive lifts with the same leg), occasionally incorporating simple arm swings at shoulder height to accentuate cadence. At vigorous intensity, movements emphasized larger ranges of motion, including power knee lifts, side kicks, and fast repeater steps, combined with overhead arm reaches to elevate cardiovascular demand. No jumping, twisting, or plyometric movements were included, to reduce joint stress and ensure consistent biomechanics across participants.

Throughout all sessions, participants were continuously monitored via heart-rate telemetry to confirm they remained within ±5 beats per minute of their prescribed HRR target zone. The supervising instructor provided real-time verbal cues and demonstrated correct form to maintain synchronization with the metronome or music beat. Safety stop criteria were pre-specified, including dizziness, chest discomfort, or shortness of breath out of proportion to intensity. Adherence was considered as completion of the full session protocol with maintenance of the prescribed choreography and HRR target zone under instructor supervision. No protocol deviations requiring session termination, rescheduling, or exclusion from analysis occurred.

All exercise sessions were performed in the same indoor studio under stable environmental conditions (temperature 20° C–22 °C, relative humidity 50%–60%, and no external distractions) and at the same time of day (afternoon between 15:00 and 18:00 h) for each participant, to minimize circadian and environmental influences on heart rate, hydration, and perceived exertion.

### Measurements

We scheduled all measurement and intervention sessions under consistent conditions throughout the data-collection period. Due to the high number of participants being recruited over several weeks, new people and groups were included gradually, but the space, time of day, room temperature, equipment setup, and procedural schedule were held constant. All sessions took place in the same indoor studio, maintained at about 20 °C–22 °C, with stable flooring and ventilation, and sessions for each participant were conducted at the same time of day to reduce circadian effects. Participants were instructed to avoid caffeine-containing or other stimulating/energy drinks, as well as strenuous physical exercise, during the 24 h preceding testing.

Physiological and psychological assessments were conducted at two stages: prior to the intervention sessions and during the exercise bouts, with all procedures performed under standardized laboratory conditions. Pre-session evaluations established baseline characteristics, including body composition and aerobic fitness. Body composition was assessed using multi-frequency bioelectrical impedance analysis (BIA). To reduce variability, BIA measurements were performed in the morning (08:00–11:00 h), in a fasted state (minimum 8 h since last meal), after voiding, and with participants instructed to abstain from caffeine, alcohol, and vigorous exercise for at least 12 h. Four psychological scales were also filled out. Cardiorespiratory fitness was determined with the Cooper 12-min run test. During the intervention sessions, continuous heart-rate monitoring was used as an objective index of internal load. Heart-rate monitors were synchronized before each session and data were logged at 1-s intervals. Perceived exertion was assessed immediately at the end of each exercise bout.

An expanded version of the instruments used can be observed in Supplementary material.

### Anthropometric and body composition procedures

Stature was measured barefoot using a wall-mounted stadiometer (Hongxing SH-2A stadiometer, Shanghai Hongxing Medical Instruments, Shanghai, China). Central adiposity was indexed by waist circumference using a non-elastic tape with participants standing, abdomen relaxed, and arms at the sides; the measurement site was taken at the midpoint between the inferior margin of the last rib and the iliac crest, consistent with contemporary clinical guidance that recognizes this landmark or the iliac-crest level as acceptable standards for monitoring risk related to abdominal adiposity (Ross et al., 2020).

Whole-body composition was then estimated with standing BIA, performed in duplicate and averaged. For the standing BIA device, we employed the Body Fat Scales (Huawei Technologies Co., Ltd., China). In our study, participants stood barefoot with heels aligned per manufacturer instructions; metal objects were removed; feet were lightly moistened with an alcohol wipe if skin was very dry; and two consecutive readings within pre-set agreement limits (±0.5 kg mass and ±0.5% body fat) were required, with a third measurement taken if limits were exceeded.

From the anthropometric and body composition procedures, the following outcomes were extracted for subsequent analyses: stature (cm), body mass (kg), and waist circumference (cm) as indicators of general and central anthropometry. Derived indices included body mass index (BMI, kg·m^−2^) and waist-to-height ratio (WHtR). From the BIA, we obtained total body fat mass (kg), and skeletal muscle mass (SMM, kg).

### Cooper 12-min run test

Cardiorespiratory fitness and maximal heart rate were assessed with the Cooper 12-min run test, performed on a 400-m outdoor track marked at 50-m intervals. In our analyses, VO₂max was estimated from distance using the published Cooper distance-based equation; we also confirmed conclusions with a population-specific regression from an external validation study in university students, which reported high correlations with directly measured VO₂max and good cross-validation performance (Bandyopadhyay, 2014). Concurrently, we derived maximal heart rate (HRmax) from the highest rolling 5-s average recorded during the 12-min run.

### Exercise self-efficacy (EXSE)

Exercise self-efficacy was assessed with the Exercise Self-Efficacy Scale [EXSE; (McAuley, 1993)], a brief instrument indexing confidence to maintain regular, moderate-intensity exercise over progressively longer time horizons. Participants rated their confidence on a 0%–100% scale in 10-point increments (0% “not at all confident” to 100% “highly confident”) for each item; scores were averaged to yield a total EXSE score (0–100).

### Behavioral inhibition/behavioral activation (BIS/BAS)

Trait sensitivity to avoidance and approach systems was measured with the BIS/BAS Scales (20 items; BIS = 7 items; BAS subdivided into Drive, Fun-Seeking, Reward Responsiveness). Items were rated on a 4-point Likert scale (“very true for me” to “very false for me”), and subscale scores were summed following standard scoring; higher scores reflect greater system sensitivity.

### Trait anxiety (STAI form Y-2)

Trait anxiety was measured with the State–Trait Anxiety Inventory, Form Y-2 (STAI-Y-2; Trait), comprising 20 items rated on a 4-point scale (“Almost Never” to “Almost Always”) with appropriate reverse-scoring; item scores were summed to a 20–80 total (higher = greater trait anxiety).

### Interoceptive sensibility (MAIA-2)

Interoceptive sensibility was assessed with the Multidimensional Assessment of Interoceptive Awareness, Version 2 (MAIA-2). The MAIA-2 contains 37 items loading on eight dimensions (Noticing, Not-Distracting, Not-Worrying, Attention Regulation, Emotional Awareness, Self-Regulation, Body Listening, Trusting), each rated on a 0–5 Likert scale (0 “never” to 5 “always”).

### Preference for and tolerance of the intensity of exercise questionnaire (PRETIE-Q)

The PRETIE-Q is a 16-item self-report scale developed to measure two distinct constructs (Ekkekakis et al., 2008; Wang et al., 2023): Preference for Intensity (how much an individual prefers higher intensity exercise) and Tolerance of Intensity (how much intensity they can tolerate). Items are rated on a 5-point Likert scale (1 = totally disagree, 5 = totally agree). Each subscale comprises 8 items, subscale scores are averaged.

### Heart rate monitoring

Heart rate (HR) was monitored continuously during all intervention sessions as the primary index of internal load. The Xiaomi Mi Band 5 (Xiaomi Corp., Beijing, China) was used. Its selection was informed by exercise-specific validation data showing good agreement with ECG at rest and during low-intensity graded exercise, although accuracy decreases as HR and exercise intensity increase (Kim et al., 2022). Raw HR time series were screened *post hoc* for implausible spikes or dropouts (e.g., abrupt changes ≥30 beats·min^−1^ within a single epoch); flagged points were corrected by linear interpolation from adjacent valid values, and any block with >10% interpolated data was flagged for sensitivity analyses. For each 3-min bout we derived minute-wise HR, end-bout HR, and block-mean %HRR (averaging the final 2 min to reflect steady-state).

### Ratings of perceived exertion (RPE)

The 6–20 Borg’s scale was verbally reported at minutes 3, 6, and 9 of the stepping bout and again immediately at bout termination; ratings were always obtained before any feedback on heart rate or performance to avoid anchoring bias. Participants were blinded to their HR values throughout the sessions, as the monitor display was not visible to them and HR information was viewed only by the research staff for safety and target-zone monitoring.

### Statistical procedures

All analyses were conducted on the long-format repeated-measures dataset, with repeated observations nested within participants across the three standardized step-aerobics sessions. Descriptive results are presented as mean ± SD for continuous variables and *n* (%) for categorical variables. The primary outcome was rating of perceived exertion (RPE, Borg 6–20), and the primary time-varying predictor was achieved heart rate reserve (HRR, %).

To test the main study objective, we fit repeated-measures generalized estimating equation (GEE) models with a Gaussian distribution, identity link, an independence working correlation structure, participant ID as the clustering variable, and robust sandwich standard errors. A base model quantified the association between achieved HRR and RPE in the pooled dataset and within each prescribed-intensity session. To evaluate moderation, each anthropometric, physiological, or psychological variable was entered in a separate model together with its main effect and the HRR × moderator interaction. Psychological scales were modeled separately rather than simultaneously because several constructs were conceptually related and potentially correlated. This was chosen to minimize multicollinearity and to preserve interpretability of the moderator-specific estimates. Session-specific models were then used to determine whether moderation patterns differed across light, moderate, and vigorous exercise.

Continuous moderators were standardized as z-scores before modeling to improve comparability across measures. Results are reported as unstandardized regression coefficients (B) with 95% confidence intervals and *p* values, together with standardized effect sizes (standardized *β*) for all main analyses. To aid interpretation, moderator main effects were expressed at 60% HRR, a practically meaningful moderate-intensity reference point. For MAIA-2, coefficients were additionally translated into the expected change in Borg-scale RPE associated with a 1-SD increase in interoceptive sensibility.

Consistent with the study’s analytic hierarchy, two exploratory analyses were additionally conducted. First, session-specific GEE models tested whether MAIA-2 was independently associated with perceived exertion after accounting for achieved HRR. Second, a pooled GEE model tested the three-way interaction HRR × MAIA-2 × sex to evaluate possible sex-specific moderation. Because vigorous-session RPE values clustered near the upper bound of the Borg scale, vigorous-intensity effects were interpreted cautiously as potentially influenced by a perceptual ceiling effect. Statistical significance was set at *p* < 0.05, but no formal multiplicity correction was applied across all moderator tests because the analyses were prespecified, organized into primary physiological and secondary psychological hypothesis families, and intended to estimate moderator-specific effects rather than to support a single omnibus screening procedure. Accordingly, interpretation emphasized 95% confidence intervals in addition to nominal *p*-values, and all exploratory analyses were interpreted cautiously. All analyses were performed in Python 3.11 using statsmodels.

## Results

The analysis comprised 126 participants and 1,134 repeated observations across the three exercise sessions. Baseline participant characteristics are summarized in Table 1, and the overall experimental protocol is illustrated in Figure 1.

**Table 1:** Baseline participant characteristics.

Characteristic	Overall (*N* = 126)	Female (*n* = 63)	Male (*n* = 63)
Age [years]	26.24 ± 4.95	26.01 ± 4.64	26.49 ± 5.30
Height [cm]	174.69 ± 7.51	174.05 ± 8.14	175.41 ± 6.72
Body mass [kg]	73.25 ± 9.50	75.64 ± 8.98	70.54 ± 9.41
Waist circumference [cm]	78.6 ± 8.5	74.5 ± 7.1	82.7 ± 7.8
Body mass index [kg·m^−2^]	24.15 ± 3.86	25.13 ± 3.82	23.03 ± 3.62
Waist-to-height ratio [a.u.]	0.46 ± 0.07	0.45 ± 0.07	0.47 ± 0.06
Body fat [%]	19.49 ± 4.97	19.38 ± 5.09	19.62 ± 4.88
Fat mass [kg]	14.23 ± 3.97	14.66 ± 4.26	13.74 ± 3.58
Skeletal muscle mass [kg]	57.15 ± 8.83	59.14 ± 8.32	54.89 ± 8.93
VO_2_max [mL·kg^−1^·min^−1^]	47.13 ± 6.81	46.86 ± 6.99	47.44 ± 6.65
Cooper distance [m]	2613.02 ± 304.51	2601.06 ± 312.24	2626.61 ± 297.56
Resting heart rate [beats·min^−1^]	61.60 ± 6.99	62.09 ± 6.30	61.05 ± 7.73
Maximal heart rate [beats·min^−1^]	189.33 ± 6.03	189.25 ± 5.84	189.42 ± 6.29
Exercise self-efficacy [0–100]	63.70 ± 15.86	63.96 ± 14.84	63.41 ± 17.06
Behavioral inhibition [score]	19.43 ± 3.38	19.55 ± 3.34	19.29 ± 3.45
BAS drive [score]	11.11 ± 2.70	11.00 ± 2.66	11.24 ± 2.77
BAS fun seeking [score]	10.88 ± 2.58	11.27 ± 2.57	10.44 ± 2.53
BAS reward responsiveness [score]	14.44 ± 2.66	14.93 ± 2.66	13.90 ± 2.57
Trait anxiety [score]	45.32 ± 11.73	45.70 ± 11.82	44.88 ± 11.71
MAIA-2 [0–5]	2.90 ± 0.79	2.95 ± 0.84	2.86 ± 0.74
Intensity preference [1–5]	2.91 ± 0.72	3.02 ± 0.68	2.78 ± 0.75
Intensity tolerance [1–5]	2.83 ± 0.71	2.87 ± 0.69	2.78 ± 0.73

Values are mean ± SD. Sex groups were balanced (63 female, 63 male). BIA, bioelectrical impedance analysis; MAIA-2, Multidimensional Assessment of Interoceptive Awareness, version 2.

**Figure 2:**
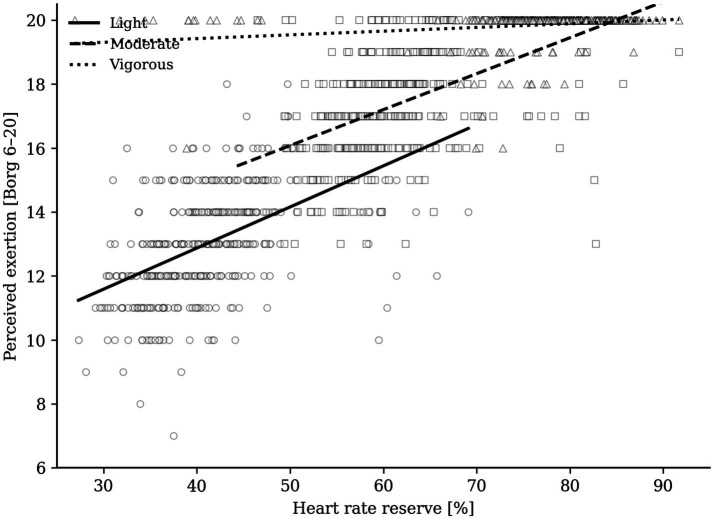
Relationship between heart rate reserve and perceived exertion across the three exercise sessions. Open symbols denote individual observations. Solid, dashed, and dotted lines are the fitted repeated-measures trends for the light, moderate, and vigorous sessions, respectively. The flattened vigorous-session slope reflects the upper-end compression of Borg ratings.

Achieved heart rate reserve (HRR) and perceived exertion increased stepwise from the light to the vigorous session (Table 2; Figure 2). Repeated-measures ANOVA on participant-level means confirmed a large effect of prescribed intensity for HRR, *F*(2, 250) = 780.09, *p* < 0.001, *η*^2^*p* = 0.862, and for perceived exertion, F(2, 250) = 1880.03, *p* < 0.001, *η*^2^*p* = 0.938; all pairwise contrasts were significant after Bonferroni correction (all *p* < 0.001). In the pooled repeated-measures model, perceived exertion increased by 0.167 Borg units for each 1% increase in HRR (95% CI [0.157, 0.176], standardized *β* = 0.859, *p* < 0.001; Table 2), indicating a large effect and confirming that relative cardiovascular load was the dominant determinant of perceived exertion in the pooled sample. However, the within-session slope was markedly attenuated in the vigorous condition (B = 0.012, 95% CI [0.002, 0.022], standardized *β* = 0.186, *p* = 0.024), indicating compression of responses near the upper end of the Borg scale.

**Table 2:** Session manipulation check and repeated-measures HRR–RPE slopes.

Condition	HRR [%]	Heart rate [beats·min^−1^]	Perceived exertion [Borg 6–20]	RPE = 20, *n* (%)	HRR → RPE slope, B [95% CI]	Std *β*	*p*
Overall	59.36 ± 16.37	137.4 ± 21.5	16.70 ± 3.17	386 (34.0%)	0.167 [0.157, 0.176]	0.859	<0.001
Light	40.79 ± 6.36	113.8 ± 9.0	12.97 ± 1.72	0 (0.0%)	0.129 [0.085, 0.172]	0.475	<0.001
Moderate	60.74 ± 6.50	139.3 ± 9.0	17.29 ± 1.67	42 (11.1%)	0.112 [0.072, 0.153]	0.439	<0.001
Vigorous	76.55 ± 8.88	159.2 ± 13.5	19.85 ± 0.56	344 (91.0%)	0.012 [0.002, 0.022]	0.186	= 0.024

The slope columns come from repeated-measures generalized estimating equation models with participant as the clustering variable. Std *β* is the fully standardized slope.

### Primary outcome: physiological moderation of the HRR–RPE association

None of the prespecified physiological moderators significantly altered the HRR–RPE slope (Table 3). Standardized interaction effects were uniformly small (absolute standardized *β* ≤ 0.047), indicating effect magnitudes that were trivial to very small in practical terms, and suggesting that cardiorespiratory fitness and body-composition indices explained little between-person variation in how perceived exertion scaled with HRR. Although the centered 60% HRR models showed modest mean-level associations for VO₂max, body mass index, waist-to-height ratio, and skeletal muscle mass, these effects were not accompanied by significant HRR × moderator interactions and therefore did not support differential scaling of perceived exertion across exercise intensity.

**Table 3:** Physiological moderators of the HRR–perceived-exertion association.

Physiological moderator	Effect at 60% HRR, B [95% CI]	*p*	HRR × moderator, B [95% CI]	Std *β* (interaction)	*p*
Cardiorespiratory fitness (VO_2_max)	−0.093 [−0.165, −0.020]	= 0.012	0.0091 [−0.0054, 0.0235]	0.047	= 0.218
Body mass index	0.082 [0.018, 0.146]	= 0.012	−0.0001 [−0.0107, 0.0105]	−0.000	= 0.987
Waist-to-height ratio	0.067 [0.005, 0.130]	= 0.034	−0.0062 [−0.0175, 0.0051]	−0.032	= 0.281
Body fat percentage	−0.053 [−0.123, 0.017]	= 0.141	−0.0008 [−0.0079, 0.0064]	−0.004	= 0.833
Fat mass	−0.003 [−0.083, 0.078]	= 0.949	−0.0020 [−0.0090, 0.0051]	−0.010	= 0.586
Skeletal muscle mass	0.093 [0.033, 0.154]	= 0.003	−0.0042 [−0.0133, 0.0049]	−0.022	= 0.368

Each moderator was analyzed in a separate repeated-measures generalized estimating equation model. Moderators were standardized as z-scores. HRR was centered at 60%, so the first effect column represents the expected change in Borg rating for a 1-SD higher moderator value at approximately moderate intensity.

### Secondary outcome: psychological moderation of the HRR–RPE association

Psychological interaction effects were likewise small (Table 4). The largest estimates were observed for interoceptive sensibility (MAIA-2; B = −0.0078 RPE points per 1% HRR per 1-SD higher score, 95% CI [−0.0160, 0.0003], standardized *β* = −0.040, *p* = 0.060) and BAS fun seeking (B = 0.0095, 95% CI [−0.0001, 0.0192], standardized *β* = 0.049, *p* = 0.053), but neither reached the conventional alpha threshold in the pooled models. Even these largest estimates were small in magnitude, indicating limited practical impact on RPE at a given HRR. All remaining psychological interactions were negligible, with absolute standardized *β* values ≤ 0.033.

**Table 4:** Psychological moderators of the HRR–perceived-exertion association.

Psychological moderator	Effect at 60% HRR, B [95% CI]	*p*	HRR × moderator, B [95% CI]	Std *β* (interaction)	*p*
Exercise self-efficacy	0.030 [−0.039, 0.099]	= 0.394	0.0002 [−0.0130, 0.0134]	0.001	= 0.975
Behavioral inhibition	−0.002 [−0.070, 0.066]	= 0.949	0.0052 [−0.0063, 0.0167]	0.027	= 0.373
BAS drive	0.039 [−0.027, 0.106]	= 0.249	0.0054 [−0.0059, 0.0167]	0.028	= 0.352
BAS fun seeking	−0.014 [−0.089, 0.060]	= 0.709	0.0095 [−0.0001, 0.0192]	0.049	= 0.053
BAS reward responsiveness	0.075 [0.005, 0.146]	= 0.036	−0.0031 [−0.0144, 0.0082]	−0.016	= 0.592
Trait anxiety (STAI-Y2)	0.015 [−0.056, 0.086]	= 0.672	0.0040 [−0.0059, 0.0140]	0.021	= 0.427
Interoceptive sensibility (MAIA-2)	−0.053 [−0.121, 0.015]	= 0.127	−0.0078 [−0.0160, 0.0003]	−0.040	= 0.060
Intensity preference	0.008 [−0.058, 0.073]	= 0.822	0.0064 [−0.0050, 0.0177]	0.033	= 0.272
Intensity tolerance	−0.033 [−0.108, 0.043]	= 0.401	0.0019 [−0.0070, 0.0108]	0.010	= 0.669

Each moderator was analyzed in a separate repeated-measures generalized estimating equation model. Moderators were standardized as z-scores, and HRR was centered at 60%.

### Exploratory analyses: MAIA-2, sex moderation, and perceptual ceiling

**Figure 3:**
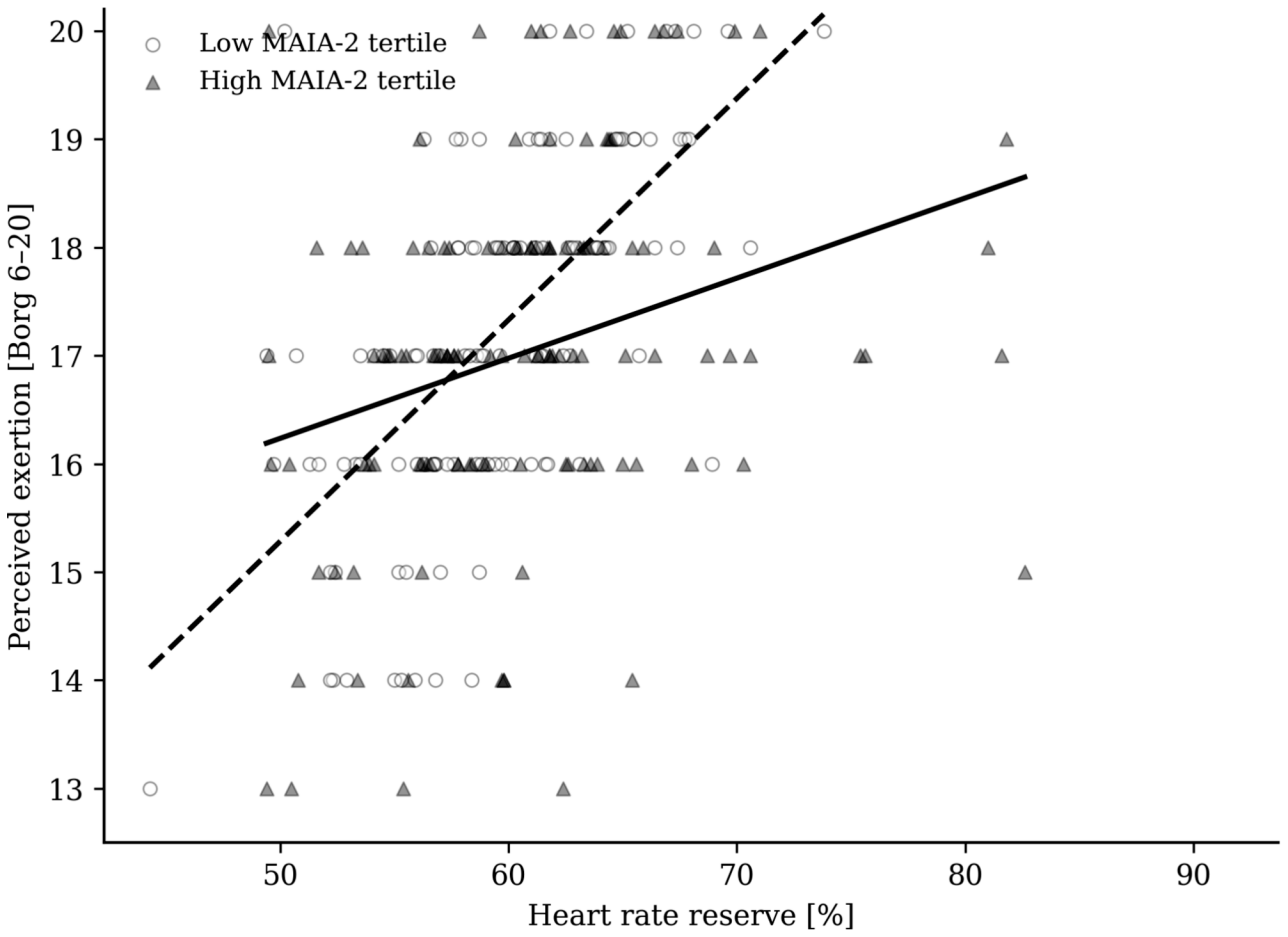
Moderate-session perceived exertion as a function of heart rate reserve in participants with low versus high MAIA-2 scores. High and low groups denote the upper and lower participant tertiles of MAIA-2. The lower fitted line for the high-MAIA group visualizes the small attenuation of perceived exertion during the moderate session.

When the models were stratified by prescribed intensity, higher MAIA-2 was associated with lower perceived exertion during the moderate session after adjustment for achieved HRR (B = −0.176, 95% CI [−0.348, −0.003], standardized *β* = −0.106, *p* = 0.046; Table 5; Figure 3). In practical terms, a 1-SD increase in MAIA-2 corresponded to an approximately 0.18-point lower Borg rating at about 60% HRR, which represents a small absolute difference in perceived exertion despite reaching nominal statistical significance in this exploratory model. Corresponding MAIA-2 effects were not evident in the light or vigorous sessions (Table 5). Exploratory repeated-measures generalized estimating equation revealed that the pooled HRR × MAIA-2 × sex interaction did not support sex-specific moderation (B = 0.011, 95% CI [−0.004, 0.026], standardized *β* = 0.058, *p* = 0.137; Table 5). At vigorous intensity, 344 of 378 observations (91.0%) were scored as 20 and 96.0% were at least 19 (Table 2), indicating a pronounced perceptual ceiling. This compression of between-person variability likely reduced sensitivity to detect moderation effects in the vigorous condition despite the clear physiological separation between sessions (Figure 2). Thus, relative internal load was the dominant determinant of perceived exertion, whereas physiological moderators were negligible and psychological moderation was limited to a small MAIA-related attenuation at moderate intensity.

**Table 5:** Exploratory MAIA-2 and sex-specific models.

Exploratory model	B [95% CI]	Std *β*	*p*
Light session: MAIA-2 effect	−0.021 [−0.157, 0.115]	−0.012	= 0.762
Moderate session: MAIA-2 effect	−0.176 [−0.348, −0.003]	−0.106	= 0.046
Vigorous session: MAIA-2 effect	−0.006 [−0.060, 0.048]	−0.011	= 0.828
Overall GEE: HRR × MAIA-2 × sex	0.011 [−0.004, 0.026]	0.058	= 0.137

The first three rows summarize session-specific MAIA-2 models adjusted for achieved HRR. The final row reports the reviewer-requested pooled repeated-measures generalized estimating equation testing the HRR × MAIA-2 × sex interaction.

## Discussion

Perceived exertion (RPE, Borg 6–20) was strongly related to relative cardiovascular load indexed by achieved HRR, confirming that %HRR was the dominant determinant of how hard the step-aerobics sessions felt in this sample. Contrary to our *a priori* expectations, however, no physiological moderator (VO₂max, BMI, WHtR, body-fat percentage, fat mass, or skeletal muscle mass) consistently altered the HRR–RPE relationship in the pooled analyses or within session-specific models. By contrast, the psychological effects were generally small, with the clearest signal involving MAIA-2, where higher interoceptive sensibility was associated with slightly lower RPE during the moderate-intensity session after adjustment for achieved HRR. These findings are consistent with the interpretation that, once step-aerobics is standardized by relative internal load, between-person differences in fitness and adiposity may contribute limited additional explanatory value for RPE. However, this interpretation should not be viewed as exclusively confirmatory, because the null physiological interactions may also reflect indirect measurement error in body composition, field-estimated cardiorespiratory fitness, and heart-rate monitoring, as well as modality-specific HR–VO₂ decoupling during step-aerobics, in which arm movements and choreographic features can elevate HR disproportionately relative to oxygen uptake. Accordingly, the absence of significant physiological moderation may reflect a combination of effective HRR standardization and residual measurement- or mode-specific noise that attenuated very small moderator effects.

A key interpretive point is that our original hypothesis that fitness and adiposity would moderate the HRR–RPE slope was theoretically plausible, but stronger for exercise prescribed at a common external workload than for exercise normalized by relative internal load. Relative-intensity prescription is designed to produce a more comparable internal physiological stress across individuals who differ in absolute exercise capacity (Mann et al., 2013). That logic helps explain why many physiological models would predict less between-person variation in perceived effort once intensity is anchored to %HRR or another relative index, even though complete equalization should not be assumed (Mann et al., 2013). We nevertheless expected some residual moderation because a choreographed stepping task may not be fully captured by heart-rate normalization alone. Individuals with higher fitness may differ in movement economy or tolerance for cardiorespiratory sensations, while individuals with greater adiposity may still experience greater local mechanical or thermal burden at a given achieved %HRR. In the present data, however, any such residual differences were too small to meaningfully alter the relative HRR–RPE slope. Although the final sample slightly exceeded the *a priori* target for the primary interaction tests, the study was powered for a conservative small interaction effect rather than for extremely small moderator effects. Accordingly, the null physiological findings argue against meaningful moderation, but they do not completely exclude the possibility of very small interaction effects that the present design may have been underpowered to detect, especially in session-specific analyses and under vigorous-session ceiling compression.

This interpretation also helps explain why the null physiological findings should not be viewed simply as failed hypotheses. Rather, they are broadly consistent with the possibility that the HRR-based standardization procedure reduced meaningful between-person physiological variance, although indirect measurement and HR–VO₂ decoupling in step-aerobics may also have attenuated detectable moderator effects. By anchoring exercise intensity to each participant’s own HRrest and HRmax, the protocol likely removed much of the between-person variance that fitness and adiposity would have contributed at an absolute workload. At the same time, HR-based standardization is not identical to perfect metabolic matching, and even relative prescription methods can leave residual heterogeneity in physiological strain across individuals.

That point may be especially relevant in step-aerobics. Prior work shows that arm movements and other aerobic-dance characteristics can elevate heart rate disproportionately relative to oxygen uptake during stepping-type exercise (Darby et al., 1995). Similarly, previous step-aerobics research reported that heart rate can overestimate the metabolic cost of the session and that the correspondence between RPE and %VO₂max may be modest at lower step heights (Sutherland et al., 1999). Accordingly, some noise in the HRR-to-exertion mapping is expected in this exercise mode, which may have further attenuated small moderator effects (Lloyd et al., 2014).

The MAIA-2 finding warrants more careful interpretation. Importantly, the MAIA-2 assesses interoceptive sensibility, that is, self-reported tendencies or beliefs about noticing, interpreting, and regulating bodily sensations, rather than interoceptive accuracy measured with objective performance-based tasks such as heartbeat detection or counting (Mehling et al., 2018; Murphy et al., 2019). This distinction is important because interoceptive sensibility and interoceptive accuracy are conceptually and empirically dissociable, and they should not be treated as interchangeable constructs (Murphy et al., 2019). Our results therefore do not suggest that objectively more accurate perception of internal signals reduced exertion. Rather, they suggest that participants reporting greater interoceptive sensibility experienced slightly lower perceived exertion during moderate exercise.

The intensity-specific pattern is also informative. The association between higher interoceptive sensibility and lower RPE was evident only in the moderate session and not in the vigorous session, where ratings clustered near the upper end of the Borg scale. A reasonable interpretation is that interoceptive sensibility may matter most when exercise is sufficiently demanding to generate salient bodily cues, but not so intense that responses compress near a perceptual ceiling (Murphy et al., 2019). Under vigorous conditions, trait-like differences in appraisal may have had less opportunity to emerge because the task was already experienced as very hard by most participants.

The lack of clear moderation by self-efficacy, BIS/BAS, trait anxiety, and PRETIE-Q dimensions should also be interpreted cautiously. These constructs may still influence affective responses, exercise choice, or tolerance in other contexts, but in the present protocol their incremental contribution to momentary RPE at a given achieved %HRR appears limited. One possibility is that the structured, music-paced, and instructor-led nature of the step-aerobics sessions constrained the expression of broader dispositional differences, leaving interoceptive sensibility as the most proximal psychological correlate of exertional appraisal in this context.

Several limitations should be acknowledged. First, the sample was restricted to healthy young adults (18–35 years), which limits generalizability to older adults and other populations in whom interoceptive styles, cardiovascular dynamics, and perceived-exertion responses may differ. In addition, participants were recruited through convenience sampling, which may have introduced selection bias by preferentially enrolling individuals who were more motivated, exercise-inclined, or accessible within university/community fitness context. Accordingly, the findings should be generalized cautiously beyond similar healthy volunteer populations. Second, several physiological constructs were assessed indirectly since body composition was estimated by bioelectrical impedance, aerobic fitness was derived from the Cooper 12-min run test rather than direct gas-exchange measurement, and internal load was indexed primarily by heart rate rather than a broader panel of physiological markers. Cardiorespiratory fitness was determined with the Cooper 12-min run test, which provides a practical field-based estimate of VO₂max but is inherently less precise than direct breath-by-breath gas analysis obtained during laboratory cardiopulmonary exercise testing. Although these approaches are practical and ecologically relevant for field-based exercise research, they introduce measurement error and may attenuate associations with perceived exertion. In addition, the psychological moderators were assessed exclusively by self-report questionnaires, which may be influenced by social-desirability bias, response styles, recall limitations, and shared method variance, thereby introducing additional measurement error into the trait estimates. In addition, heart rate in step-aerobics may be elevated disproportionately relative to oxygen uptake because of arm elevation and upper-body movement, introducing modality-specific HR–VO₂ decoupling. Finally, participants were tested under standardized environmental and time-of-day conditions, but female hormonal status was not specifically controlled since menstrual cycle phase and hormonal contraceptive use were not standardized in the design and could have influenced heart-rate and perceived-exertion responses, adding unexplained within- and between-participant variability. More specifically, fluctuations in estrogen and progesterone across the menstrual cycle can alter thermoregulatory, ventilatory, and cardiovascular responses to exercise, and may in some individuals also shift perceived exertion. Therefore, lack of cycle control may have introduced additional noise into both HRR and RPE, potentially confusing very small moderator effects. An additional limitation is that RPE was analyzed primarily with Gaussian GEE despite the Borg 6–20 scale being ordinal and vigorous-session responses showing evident clustering at the upper bound. Although this approach provided a consistent population-averaged framework across all sessions, alternative models such as cumulative-link ordinal regression or censored repeated-measures models may better accommodate ordered responses and ceiling compression, and should be considered in future sensitivity analyses.

From a practical standpoint, the present findings directly support the use of HRR-based prescription for mixed-ability aerobics groups, because perceived exertion increased in a broadly predictable way once exercise was standardized by relative internal load. This evidence-based implication is stronger than any conclusion regarding psychological tailoring. The interoceptive sensibility result should be interpreted more cautiously, because it was small and limited to exploratory analyses. Accordingly, it is reasonable only to hypothesize that how people attend to and regulate bodily sensations may influence the subjective experience of moderate exercise. Implications for enjoyment, adherence, or long-term exercise behavior remain hypothetical, because these outcomes were not measured directly. Future studies should test, rather than assume, whether interventions designed to enhance adaptive interoceptive sensibility (such as non-judgmental body awareness, breathing-focused attention, or pacing strategies) can reduce perceived exertion, improve affective responses, or support adherence over time.

## Conclusion

In this study of healthy young adults performing standardized, HRR-calibrated step-aerobics, perceived exertion scaled robustly with relative cardiovascular load, but was not significantly moderated by aerobic fitness, BMI, central adiposity, fat mass, or skeletal muscle mass. Instead, interoceptive sensibility (MAIA-2) consistently emerged as the most salient correlate of lower RPE at light and moderate intensities, with effects dissipating under vigorous workloads where perceptual ceilings constrained variability. These findings highlight that, when exercise intensity is prescribed relatively, psychological dispositions toward bodily awareness may explain individual differences in how effort is perceived more strongly than physiological or body-composition status. Accordingly, in healthy young adults performing step-aerobics, HRR appears to be the dominant determinant of perceived exertion, whereas generalization beyond similar populations should be made cautiously. Practically, the clearest implication is that HRR-based prescription may help standardize exertional load in mixed-ability step-aerobics settings, whereas any added value of interoceptive-awareness strategies remains hypothetical and requires direct testing. Future research should extend this work across ages, clinical groups, and modalities, and test whether training interoceptive skills can sustainably reshape exertional experiences and exercise adherence.

## Data Availability

The raw data supporting the conclusions of this article will be made available by the authors, without undue reservation.
