# Supplementary material for: Psychological and physiological moderators of perceived exertion in aerobics: a repeated-measures study
Source: Front Psychol. 2026 May 7;17:1719976. doi: 10.3389/fpsyg.2026.1719976 (PMC13189887; doi:10.3389/fpsyg.2026.1719976)
Supplement: Supplementary file 1 [file Supplementary_file_1.docx]

**Supplementary material 1.** Expanded version of measurements.

*Measurements*

We scheduled all measurement and intervention sessions under consistent conditions throughout the data‐collection period. Due to the high number of participants being recruited over several weeks, new people and groups were included gradually, but the space, time of day, room temperature, equipment setup, and procedural schedule were held constant. All sessions took place in the same indoor studio, maintained at about 20–22 °C, with stable flooring and ventilation, and sessions for each participant were conducted at the same time of day to reduce circadian effects. Participants were instructed to avoid caffeine-containing or other stimulating/energy drinks, as well as strenuous physical exercise, during the 24 h preceding testing.

Physiological and psychological assessments were conducted at two stages: prior to the intervention sessions and during the exercise bouts, with all procedures performed under standardized laboratory conditions. Pre-session evaluations established baseline characteristics, including body composition and aerobic fitness. Body composition was assessed using multi-frequency bioelectrical impedance analysis (BIA). To reduce variability, BIA measurements were performed in the morning (08:00–11:00 h), in a fasted state (minimum 8 h since last meal), after voiding, and with participants instructed to abstain from caffeine, alcohol, and vigorous exercise for at least 12 h. Four psychological scales were also filled out. Cardiorespiratory fitness was determined with the Cooper 12-minute run test. During the intervention sessions, continuous heart-rate monitoring was used as an objective index of internal load). Heart-rate monitors were synchronized before each session and data were logged at 1-second intervals. Perceived exertion was assessed immediately at the end of each exercise bout.

*Anthropometric and Body Composition Procedures*

Stature was measured barefoot using a wall-mounted stadiometer (Hongxing SH-2A stadiometer, Shanghai Hongxing Medical Instruments, Shanghai, China). Central adiposity was indexed by waist circumference using a non-elastic tape with participants standing, abdomen relaxed, and arms at the sides; the measurement site was taken at the midpoint between the inferior margin of the last rib and the iliac crest, consistent with contemporary clinical guidance that recognizes this landmark or the iliac-crest level as acceptable standards for monitoring risk related to abdominal adiposity (Ross et al., 2020).

Whole-body composition was then estimated with standing BIA, performed in duplicate and averaged. To minimize sources of error known to influence impedance—recent fluid or food intake, body position, skin temperature, and prior exercise—participants arrived euhydrated, voided, and refrained from alcohol, caffeine, and vigorous activity for ≥12 h; measurements were scheduled consistently (late morning/afternoon for the cohort) and conducted in a thermo-neutral room. These procedures align with best-practice recommendations and reviews noting that standardized preparation and posture improve the precision and interpretability of BIA-derived fat-free mass and fat mass estimates (Kyle, 2004). Recent experimental work further underscores the relevance of testing posture and hydration control for standing BIA devices, with demonstrable differences between lying vs standing positions and with acute fluid intake, supporting our choice to fix measurement position and pre-measurement instructions (Więch et al., 2022). Although some data suggest that a standardized light breakfast may have minimal practical impact on certain BIA outcomes, research guidelines still recommend longer fasting windows for research standardization; thus, our protocol maintained conservative abstention periods to reduce variability (Korzilius et al., 2023).

For the standing BIA device, we employed the Body Fat Scales (Huawei Technologies Co., Ltd., China). In a prospective comparison against a clinical tetrapolar analyzer and dual-energy X-ray absorptiometry (DXA), the Huawei scale—tested in both standard 4-electrode foot-to-foot mode and an 8-electrode configuration with a retractable handgrip—showed very good intra- and inter-unit precision and small, systematic biases relative to DXA (fat-mass percentage accuracy ±3.3–3.4% for standing modes), supporting its suitability for rapid body-composition assessment in non-bedridden adults when used under standardized conditions (Hamilton-James et al., 2021). In our study, participants stood barefoot with heels aligned per manufacturer instructions; metal objects were removed; feet were lightly moistened with an alcohol wipe if skin was very dry; and two consecutive readings within pre-set agreement limits (±0.5 kg mass and ±0.5 % body fat) were required, with a third measurement taken if limits were exceeded.

From the anthropometric and body composition procedures, the following outcomes were extracted for subsequent analyses: stature (cm), body mass (kg), and waist circumference (cm) as indicators of general and central anthropometry. Derived indices included body mass index (BMI, kg·m⁻²) and waist-to-height ratio (WHtR). From the BIA, we obtained total body fat mass (kg), and skeletal muscle mass (SMM, kg).

*Cooper 12-minute run test*

Cardiorespiratory fitness and maximal heart rate were assessed with the Cooper 12-minute run test, performed on a 400-m outdoor track marked at 50-m intervals. After a standardized warm-up, participants were instructed to cover the greatest possible distance in 12 minutes at a self-paced, maximal sustainable effort; lap counts and final distance (nearest 10 m) were recorded by two observers. The Cooper test is a classic field protocol introduced by Cooper, who demonstrated strong associations between 12-minute distance and laboratory VO₂max obtained on a treadmill, establishing the test’s criterion validity for estimating aerobic capacity in adults (Cooper, 1968). In our analyses, VO₂max was estimated from distance using the published Cooper distance-based equation; we also confirmed conclusions with a population-specific regression from an external validation study in university students, which reported high correlations with directly measured VO₂max and good cross-validation performance (Bandyopadhyay, 2014).

Concurrently, we derived maximal heart rate (HRmax) from the highest rolling 5-s average recorded during the 12-minute run. Using continuous maximal field runs to obtain HRmax is supported by comparisons showing that peak HR achieved in well-controlled field running tests is similar to, and sometimes not statistically different from, values elicited during incremental treadmill cardiopulmonary tests, whereas modality changes (e.g., swimming) can yield lower peaks than running—underscoring the importance of using modality-specific maximal tests for prescription (DiCarlo et al., 1991; Bennett et al., 2024). We used this measured HRmax (rather than age-predicted formulas) to individualize session intensities via the Karvonen heart-rate reserve method, given extensive evidence that age-predicted equations (e.g., 220–age) show large individual errors and systematic bias relative to measured HRmax (Tanaka et al., 2001).

*Exercise Self-Efficacy (EXSE)*

Exercise self-efficacy was assessed with the Exercise Self-Efficacy Scale (EXSE; (McAuley, 1993)), a brief instrument indexing confidence to maintain regular, moderate-intensity exercise over progressively longer time horizons. Participants rated their confidence on a 0–100% scale in 10-point increments (0% “not at all confident” to 100% “highly confident”) for each item; scores were averaged to yield a total EXSE score (0–100). The EXSE has been used extensively in exercise research with excellent internal consistency and predictive validity for adherence; we followed the standard wording and scoring conventions described in McAuley’s program of work and subsequent applications (McAuley et al., 2011; Phillips et al., 2013). Administration occurred at baseline prior to any fitness testing.

*Behavioral Inhibition/Behavioral Activation (BIS/BAS)*

Trait sensitivity to avoidance and approach systems was measured with the BIS/BAS Scales (20 items; BIS = 7 items; BAS subdivided into Drive, Fun-Seeking, Reward Responsiveness). Items were rated on a 4-point Likert scale (“very true for me” to “very false for me”), and subscale scores were summed following standard scoring; higher scores reflect greater system sensitivity. The BIS/BAS has well-documented factor structure and reliability across clinical and non-clinical samples (Campbell-Sills et al., 2004); we used the conventional four-factor solution and computed BIS and the three BAS subscales for moderation analyses.

*Trait Anxiety (STAI Form Y-2)*

Trait anxiety was measured with the State–Trait Anxiety Inventory, Form Y-2 (STAI-Y-2; Trait), comprising 20 items rated on a 4-point scale (“Almost Never” to “Almost Always”) with appropriate reverse-scoring; item scores were summed to a 20–80 total (higher = greater trait anxiety). We used contemporary psychometric work to inform interpretation in non-clinical adult samples and to support the scale’s criterion validity and diagnostic accuracy benchmarks (Ilardi et al., 2021; Shah et al., 2021).

*Interoceptive Sensibility (MAIA-2)*

Interoceptive sensibility was assessed with the Multidimensional Assessment of Interoceptive Awareness, Version 2 (MAIA-2). The MAIA-2 contains 37 items loading on eight dimensions (Noticing, Not-Distracting, Not-Worrying, Attention Regulation, Emotional Awareness, Self-Regulation, Body Listening, Trusting), each rated on a 0–5 Likert scale (0 “never” to 5 “always”). Dimension scores were computed as item means (higher = greater interoceptive sensibility on that facet); no global total was analyzed. The MAIA-2 shows improved internal consistency over the original MAIA, with confirmatory factor analyses supporting its 8-factor structure (Mehling et al., 2018).

*Preference for and Tolerance of the Intensity of Exercise Questionnaire (PRETIE-Q)*

The PRETIE-Q is a 16-item self-report scale developed to measure two distinct constructs (Ekkekakis et al., 2008; Wang et al., 2023): Preference for Intensity (how much an individual prefers higher intensity exercise) and Tolerance of Intensity (how much intensity they can tolerate). Items are rated on a 5-point Likert scale (1 = totally disagree, 5 = totally agree). Each subscale comprises 8 items, subscale scores are averaged. In its original validation among college women (n = 601), internal consistency was high (α = .89 for Preference; α = .86 for Tolerance), and confirmatory factor analysis supported the two-factor structure (Ekkekakis et al., 2008).

*Reliability of the psychological instruments*

Pilot testing was conducted in 10% of the sample to assess instrument reliability. Internal consistency was excellent for the Exercise Self-Efficacy Scale (EXSE; Cronbach’s α = 0.95 across repeated assessments), high for the STAI-Y2 trait scale (α = 0.92), good for the MAIA-2 (α = 0.83) and PRETIE-Q (α = 0.89), and acceptable for the BIS/BAS Scales (BIS: α = 0.74; BAS-Drive: α = 0.76; BAS-Reward Responsiveness: α = 0.73; BAS-Fun Seeking: α = 0.72).

*Heart Rate Monitoring*

Heart rate (HR) was monitored continuously during all intervention sessions as the primary index of internal load. Participants wore the device on the non-dominant wrist, with the strap fitted snugly to minimize motion artifact, and were instructed to avoid unnecessary wrist motions and talking during the bouts. A 5-minute seated baseline was recorded before the warm-up to obtain resting HR. During the 9-minute stepping bout, HR was recorded at the device’s native sampling frequency in workout mode and exported in 5-second epochs for analysis; during the subsequent 3-minute seated recovery, HR was recorded continuously. The Karvonen heart-rate reserve (HRR) method was used to individualize intensity targets: %HRR = $(\text{HR}_{\text{exercise}}-\text{HR}_{\text{rest}})/(\text{HR}_{\text{max}}-\text{HR}_{\text{rest}})\times100$, with $\mathrm{HR}_{\text{max}}$ taken as the highest heart rate reached during the Cooper 12-minute run test. This approach is based in the classic Karvonen work and subsequent applications linking %HRR to aerobic training zones (Scharff-Olson et al., 1992a).

The Xiaomi Mi Band 5 (Xiaomi Corp., Beijing, China) was used. In cardiopulmonary exercise testing, the Mi Band 5 displayed high agreement with ECG at rest and low intensities (Kim et al., 2022). Raw HR time series were screened post hoc for implausible spikes or dropouts (e.g., abrupt changes ≥30 beats·min⁻¹ within a single epoch); flagged points were corrected by linear interpolation from adjacent valid values, and any block with >10% interpolated data was flagged for sensitivity analyses. For each 3-minute bout we derived minute-wise HR, end-bout HR, and block-mean %HRR (averaging the final 2 minutes to reflect steady-state). These procedures align with prior evidence showing strong HR–VO₂ coupling in stepping/aerobic dance and support the use of HRR as an individualized internal-load anchor for interpreting perceived exertion responses across light, moderate, and vigorous sessions (Scharff-Olson et al., 1992b).

*Ratings of Perceived Exertion (RPE)*

Before the first session, participants received standardized instructions adapted from Borg’s original guidance: they were shown a laminated 6–20 scale with verbal and numeric anchors, told to rate their overall (global) sense of effort rather than local muscle discomfort or breathlessness alone, and completed a brief practice bout (marching/stepping) to calibrate responses. During the experimental sessions, the supervising researcher held the laminated scale at eye level and asked the standardized question: “How hard does the exercise feel right now?”. The RPE was verbally reported at minutes 3, 6, and 9 of the stepping bout and again immediately at bout termination; ratings were always obtained before any feedback on heart rate or performance to avoid anchoring bias. The Borg 6–20 scale’s psychophysical basis and intended use are described in Borg’s seminal paper (Borg, 1982), and its criterion validity is supported by meta-analytic evidence across healthy adults (Chen et al., 2002). Reliability of repeated RPE sampling under standardized procedures is well documented, supporting its use in repeated-measures protocols like ours (Eston and Williams, 1988).

References

Bandyopadhyay, A. (2014). Validity of Cooper’s 12-minute run test for estimation of maximum oxygen uptake in male university students. *Biol. Sport* 32, 59–63. doi: 10.5604/20831862.1127283

Bennett, T., Marshall, P., Barrett, S., Malone, J. J., Simpson, A., Bray, J., et al. (2024). Validation of field-based running tests to determine maximal aerobic speed in professional rugby league. *PLoS One* 19, e0306062. doi: 10.1371/journal.pone.0306062

Borg, G. A. V. (1982). Psychophysical bases of perceived exertion. *Med. Sci. Sports Exerc.* 14, 377–381. doi: 10.1249/00005768-198205000-00012

Campbell-Sills, L., Liverant, G. I., and Brown, T. A. (2004). Psychometric Evaluation of the Behavioral Inhibition/Behavioral Activation Scales in a Large Sample of Outpatients With Anxiety and Mood Disorders. *Psychol. Assess.* 16, 244–254. doi: 10.1037/1040-3590.16.3.244

Chen, M. J., Fan, X., and Moe, S. T. (2002). Criterion-related validity of the Borg ratings of perceived exertion scale in healthy individuals: a meta-analysis. *J. Sports Sci.* 20, 873–899. doi: 10.1080/026404102320761787

Cooper, K. H. (1968). A means of assessing maximal oxygen intake. Correlation between field and treadmill testing. *JAMA* 203, 201–4.

DiCarlo, L., Sparling, P., Millard-Stafford, M., and Rupp, J. (1991). Peak Heart Rates during Maximal Running and Swimming: Implications for Exercise Prescription. *Int. J. Sports Med.* 12, 309–312. doi: 10.1055/s-2007-1024687

Ekkekakis, P., Thome, J., Petruzzello, S. J., and Hall, E. E. (2008). The Preference for and Tolerance of the Intensity of Exercise Questionnaire: A psychometric evaluation among college women. *J. Sports Sci.* 26, 499–510. doi: 10.1080/02640410701624523

Eston, R. G., and Williams, J. G. (1988). Reliability of ratings of perceived effort regulation of exercise intensity. *Br. J. Sports Med.* 22, 153–155. doi: 10.1136/bjsm.22.4.153

Hamilton-James, K., Collet, T.-H., Pichard, C., Genton, L., and Dupertuis, Y. M. (2021). Precision and accuracy of bioelectrical impedance analysis devices in supine versus standing position with or without retractable handle in Caucasian subjects. *Clin. Nutr. ESPEN* 45, 267–274. doi: 10.1016/j.clnesp.2021.08.010

Ilardi, C. R., Gamboz, N., Iavarone, A., Chieffi, S., and Brandimonte, M. A. (2021). Psychometric properties of the STAI-Y scales and normative data in an Italian elderly population. *Aging Clin. Exp. Res.* 33, 2759–2766. doi: 10.1007/s40520-021-01815-0

Kim, C., Kim, S. H., and Suh, M. R. (2022). Accuracy and Validity of Commercial Smart Bands for Heart Rate Measurements During Cardiopulmonary Exercise Test. *Ann. Rehabil. Med.* 46, 209–218. doi: 10.5535/arm.22050

Korzilius, J. W., Oppenheimer, S. E., de Roos, N. M., Wanten, G. J. A., and Zweers, H. (2023). Having breakfast has no clinically relevant effect on bioelectrical impedance measurements in healthy adults. *Nutr. J.* 22, 55. doi: 10.1186/s12937-023-00882-5

Kyle, U. (2004). Bioelectrical impedance analysis?part I: review of principles and methods. *Clinical Nutrition* 23, 1226–1243. doi: 10.1016/j.clnu.2004.06.004

McAuley, E. (1993). Self-efficacy and the maintenance of exercise participation in older adults. *J. Behav. Med.* 16, 103–113. doi: 10.1007/BF00844757

McAuley, E., Mailey, E. L., Mullen, S. P., Szabo, A. N., Wójcicki, T. R., White, S. M., et al. (2011). Growth trajectories of exercise self-efficacy in older adults: Influence of measures and initial status. *Health Psychology* 30, 75–83. doi: 10.1037/a0021567

Mehling, W. E., Acree, M., Stewart, A., Silas, J., and Jones, A. (2018). The Multidimensional Assessment of Interoceptive Awareness, Version 2 (MAIA-2). *PLoS One* 13, e0208034. doi: 10.1371/journal.pone.0208034

Phillips, S. M., Wójcicki, T. R., and McAuley, E. (2013). Physical activity and quality of life in older adults: an 18-month panel analysis. *Quality of Life Research* 22, 1647–1654. doi: 10.1007/s11136-012-0319-z

Ross, R., Neeland, I. J., Yamashita, S., Shai, I., Seidell, J., Magni, P., et al. (2020). Waist circumference as a vital sign in clinical practice: a Consensus Statement from the IAS and ICCR Working Group on Visceral Obesity. *Nat. Rev. Endocrinol.* 16, 177–189. doi: 10.1038/s41574-019-0310-7

Scharff-Olson, M., Williford, H. N., and Smith, F. H. (1992a). The heart rate VO2 relationship of aerobic dance: a comparison of target heart rate methods. *J. Sports Med. Phys. Fitness* 32, 372–7.

Scharff-Olson, M., Williford, H. N., and Smith, F. H. (1992b). The heart rate VO2 relationship of aerobic dance: a comparison of target heart rate methods. *J. Sports Med. Phys. Fitness* 32, 372–7.

Shah, N. N., Schwandt, M. L., Hobden, B., Baldwin, D. S., Sinclair, J., Agabio, R., et al. (2021). The validity of the state–trait anxiety inventory and the brief scale for anxiety in an inpatient sample with alcohol use disorder. *Addiction* 116, 3055–3068. doi: 10.1111/add.15516

Tanaka, H., Monahan, K. D., and Seals, D. R. (2001). Age-predicted maximal heart rate revisited. *J. Am. Coll. Cardiol.* 37, 153–156. doi: 10.1016/S0735-1097(00)01054-8

Wang, T., Kuang, J., Herold, F., Taylor, A., Ludyga, S., Zhang, Z., et al. (2023). Validity and Reliability of the Preference for and Tolerance of the Intensity of Exercise Questionnaire among Chinese College Students. *International Journal of Mental Health Promotion* 25, 127–138. doi: 10.32604/ijmhp.2022.022504

Więch, P., Wołoszyn, F., Trojnar, P., Skórka, M., and Bazaliński, D. (2022). Does Body Position Influence Bioelectrical Impedance? An Observational Pilot Study. *Int. J. Environ. Res. Public Health* 19, 9908. doi: 10.3390/ijerph19169908
